# Two-stage hepatectomy in resection of colorectal liver metastases – a single-institution experience with case-control matching and review of the literature

**DOI:** 10.2478/raon-2023-0026

**Published:** 2023-06-21

**Authors:** Spela Turk, Irena Plahuta, Tomislav Magdalenic, Tajda Spanring, Kevin Laufer, Zan Mavc, Stojan Potrc, Arpad Ivanecz

**Affiliations:** Clinical Department of Abdominal and General Surgery, University Medical Centre Maribor, Maribor, Slovenia; Department of Surgery, Faculty of Medicine, University of Maribor, Maribor, Slovenia

**Keywords:** colorectal cancer, liver metastases, hepatectomy, future liver remnant, posthepatectomy liver failure, survival analysis

## Abstract

**Background:**

Two-stage hepatectomy (TSH) has been proposed for patients with bilateral liver tumours who have a high risk of posthepatectomy liver failure after one-stage hepatectomy (OSH). This study aimed to determine the outcomes of TSH for extensive bilateral colorectal liver metastases.

**Patients and methods:**

A retrospective review of a prospectively maintained database of liver resections for colorectal liver metastases was conducted. The TSH group was compared to the OSH group in terms of perioperative outcomes and survival. Case-control matching was performed.

**Results:**

A total of 632 consecutive liver resections for colorectal liver metastases were performed between 2000 and 2020. The study group (TSH group) consisted of 15 patients who completed TSH. The control group included 151 patients who underwent OSH. The case-control matching-OSH group consisted of 14 patients. The major morbidity and 90-day mortality rates were 40% and 13.3% in the TSH group, 20.5% and 4.6% in the OSH group and 28.6% and 7.1% in the case-control matching-OSH group, respectively. The recurrence-free survival, median overall survival, and 3- and 5-year survival rates were 5 months, 21 months, 33% and 13% in the TSH group; 11 months, 35 months, 49% and 27% in the OSH group; and 8 months, 23 months, 36% and 21%, respectively, in the case-control matching-OSH group, respectively.

**Conclusions:**

TSH used to be a favourable therapeutic choice in a select population of patients. Now, OSH should be preferred whenever feasible because it has lower morbidity and equivalent oncological outcomes to those of completed TSH.

## Introduction

Colorectal cancer is the third most diagnosed cancer worldwide.^1^ At diagnosis, the disease has spread to the liver in 15% to 25% of patients, and another 25% develop colorectal liver metastases metachronously.^2^ Liver resection remains the only potentially curative treatment option for these patients.^2^ Despite the ability of the liver to regenerate after significant tissue loss, a future liver remnant, which contributes 25–30% of the total liver volume, has been the minimal requirement in patients with a noncirrhotic liver.^2^ Therefore, major hepatectomies are associated with a high risk of posthepatectomy liver failure.^3^ Innovative approaches have been developed to improve colorectal liver metastases resectability, i.e., two-stage hepatectomy (TSH).^2,4^ Their initial phase can be portal vein embolization or intraoperative selective portal vein ligation. The novelist approach is the associating liver partition and portal vein ligation for staged hepatectomy (ALPPS) procedure.^2^

This study aimed to determine the feasibility and safety of TSH for patients with extensive bilateral colorectal liver metastases by comparing perioperative and long-term outcomes between TSH and one-stage hepatectomy (OSH) groups.

## Patients and methods

### Study population

A retrospective review of a prospectively obtained database of 632 consecutive liver procedures for colorectal liver metastases at the Clinical Department of Abdominal and General Surgery of the University Medical Centre Maribor in Slovenia was performed. This department is a specialised referral centre for hepato-pancreato-biliary surgery. The study period was from 1 January 2000 until 31 December 2020.

Before the surgery, patients consented to their anonymous data being used for research. Therefore, their records were anonymised and deidentified before analysis. Ethical approval for this study was obtained from the National Medical Ethics Committee of the Republic of Slovenia (0120-455/2020/3). All methods were performed following the relevant guidelines and regulations.

#### Inclusion and exclusion criteria

The inclusion criteria were patients with bilateral colorectal liver metastases who:

- completed TSH or
- underwent their first OSH for colorectal liver metastases,
- the TSH group was formed from patients who underwent portal vein embolization or portal vein ligation, as proposed by Regimbeau.^5^

The exclusion criteria were as follows:

- explorative laparotomies without liver resections,
- repeated liver resections,
- patients with unilateral colorectal liver metastases,
- radiofrequency ablation (RFA) or its combinations with liver resections.

### Definitions

Routinely available clinical characteristics were analysed, including patient demographics, performance status defined according to the American Society of Anaesthesiologists Classification (ASA classification)^6^, application of neoadjuvant chemotherapy, preoperative carcinoembryonic antigen (CEA) level, and presence of extrahepatic disease. Primary colorectal tumour variables included the tumour location and nodal invasion. Liver metastasis variables included synchronous/metachronous metastases and the number and size of metastases.

Patients were presented at the multidisciplinary team meeting.^2^ Bilateral colorectal liver metastases were resected in a single procedure when both the volume and function of the future liver remnant were considered sufficient. The parenchyma-sparing principle of liver surgery for colorectal liver metastases was applied.^7^ The types of liver resections were classified according to the Brisbane terminology.^8^ Major liver resections involved three or more adjacent liver segments, including conventional major resections (left/extended left hepatectomies, right/extended right hepatectomies, central hepatectomies).^8^ The analysis of future liver remnant consisted of computed tomography (CT) volumetry, laboratory liver tests (prothrombin time and albumins), and the indocyanine green clearance test.^2^

Specimens were analysed by a gastrointestinal histopathologist who assessed the resection margin. The histological surgical margins for malignant lesions were defined as microscopically negative (R0) or positive (<1 mm, R1).^7^ In addition, the Clinical Risk Score devised by Fong *et al*. was applied.^9^

### Two-stage hepatectomy

Portal vein embolization, intraoperative selective portal vein ligation, or the ALPPS procedure were performed when the analysis suggested an insufficient future liver remnant.^2^ Portal vein embolization was followed by atrophy of the embolized hemiliver and hypertrophy of the other hemiliver.^2^ TSH with portal vein ligation was performed when the intraoperative findings were unfavourable.^4^ In the first stage, the metastasectomy of one hemiliver was performed along with portal vein ligation for the other hemiliver.^4^ The effect was similar to that of preoperative portal vein embolization.^10^ The second stage followed a few weeks later and consisted of a major hepatectomy.^2,4^ ALPPS was performed with the same rationale; the difference was the addition of liver parenchyma transection in the first stage.^11^

**FIGURE 1.**
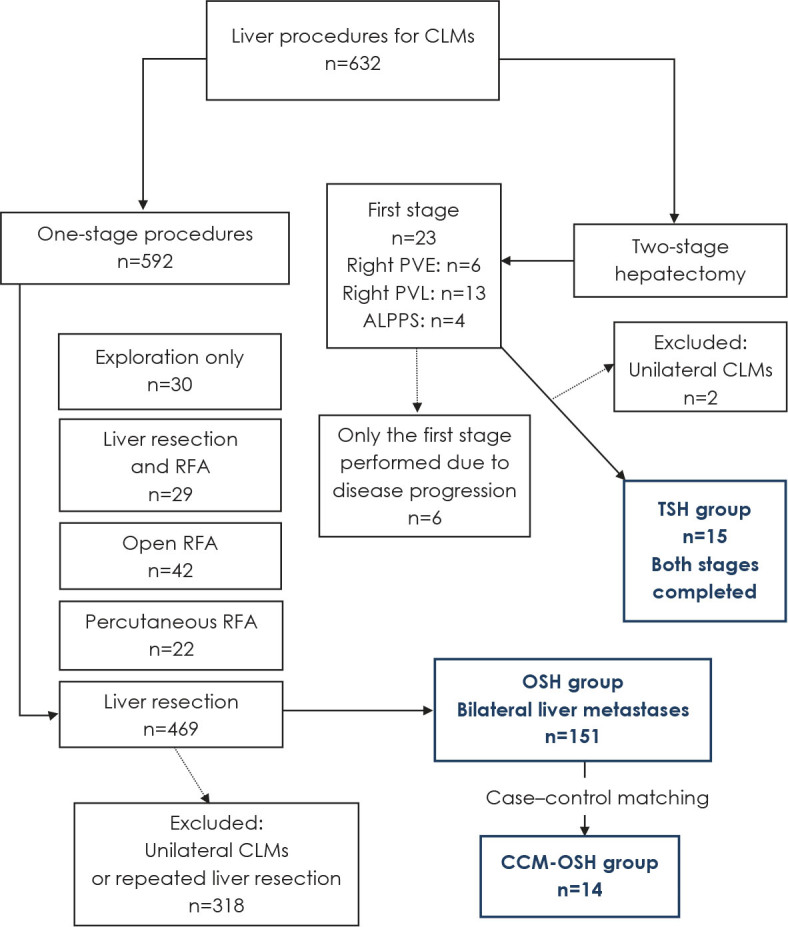
The study flowchart. The study period covers 1 January 2000 to 31 December 2020. ALPPS = associating liver partition and portal vein ligation for staged hepatectomy; CCM-OSH = case-control matching one-stage hepatectomy; CLMs = colorectal liver metastases; OSH = one-stage hepatectomy; PVE = portal vein embolization; PVL = portal vein ligation; RFA = radiofrequency ablation; TSH = two-stage hepatectomy

RFA has been applied where radical liver resection has been infeasible due to the proximity of large vessels.^2^ Therefore, RFA has been applied intraoperatively as an independent procedure or adjunct to liver resection.^2^ RFA has also been used as a percutaneous procedure. However, these patients were excluded from the analyses.

### Follow-up

Patients were followed-up at the outpatient clinic at periodic intervals. The follow-up protocol consisted of a CEA level, a chest radiograph or CT, an abdominal ultrasound, CT, or magnetic resonance imaging every three months for the first two years and every six months afterwards.^2^

### Study endpoints

#### Primary outcomes – overall survival and recurrence-free survival

The primary outcome was overall survival (OS). It was defined as the interval between the date of liver resection (the second stage in the TSH group) of colorectal liver metastases and the date of death or the last follow-up in surviving patients.

The second primary outcome was recurrence-free survival (RFS). It was calculated from the date of liver resection (the second stage in the TSH group) to the date of any detected recurrence or the last follow-up in patients without recurrence.

#### Secondary outcomes – morbidity and mortality

Morbidity was reported according to the Clavien-Dindo (CD) classification.^12^ Major morbidity was defined as CD ≥ 3a. Mortality rates were reported as the number of patients who died within 90 postoperative days.

Posthepatectomy haemorrhage, bile leakage, and liver failure were graded according to the International Study Group of Liver Surgery (ISGLS).^13,14,15^

### Statistical analysis

IBM SPSS for Windows Version 28.0 (IBM Corp., Armonk, NY, USA) was used for the statistical analysis. Percentages are reported to one decimal place. A P value ≤ 0.05 was considered statistically significant.

Categorical variables are displayed as numbers with percentages. The differences between categorical variables were tested using the chi-square or Fisher-Freeman Halton test when more than two categories were present. Continuous variables were expressed as medians (minimum-maximum, interquartile range) and analysed with the Mann-Whitney test since the distribution analysis showed the non-normal distribution of data.

Survival data for median OS and RFS are presented as Kaplan-Meier curves, and groups were compared by a log-rank test. The results are expressed in months as the median (95% confidence interval (95% CI)). Survival tables were used for 3-and 5-year OS and RFS, given in percentages.

Case-control matching was performed for 15 patients from the TSH group.^16^ Patients from the OSH group (controls) were selected based on the variables that were statistically significant in a bivariate analysis. The sampling was performed without replacement and with maximising execution performance modality. Matched patients were assigned to the case-control matching-OSH group. The statistical analysis of continuous variables was performed with the Wilcoxon signed ranks test. The analysis of categorical variables was performed with the McNemar test.^16^ Survival was estimated as described previously.

## Results

The study population was stratified into two groups. The study group TSH consisted of 15 patients who completed TSH. The control group OSH included 151 patients. The study flowchart is shown in Figure 1.

### Clinical characteristics of patients

This study included 166 patients: 151 in the OSH group and 15 in the TSH group. Their clinical characteristics and perioperative outcomes are summarised in Table 1.

**TABLE 1.** Clinical characteristics and perioperative outcomes of the 166 patients

**Clinical characteristics**	**OSH (n=151)**	**TSH (n=15)**	**P value**
**Male sex ^a^**	109 (72.2%)	13 (86.7%)	0.365
**Age (years) ^b^**	62 (34–84; 14)	64 (45–75; 12)	0.819
**ASA score ≥ 3 ^a^**	32 (21.2%)	2 (13.3%)	0.701
**Primary tumour location ^c^**			
Right colon	27 (17.9%)	1 (6.7%)	0.166
Left colon	61 (40.4%)	6 (40.0%)	
Rectum	61 (40.4%)	7 (46.7%)	
> 1 primary tumour	2 (1.3%)	1 (6.7%)	
**Primary tumour nodal invasion ^a^**	99 (66.0%)	10 (66.7%)	1.000
**Synchronous liver metastases ^a^**	81 (53.6%)	11 (73.3%)	0.179
**Number of liver metastases ^b^**	3 (1–19; 3)	5 (2–12; 6)	**0.001**
**Size of liver metastases (cm) ^b^**	4 (0.6–20; 3)	5 (1.5–11; 5.5)	0.183
**Neoadjuvant chemotherapy ^a^**	79 (52.3%)	13 (86.7%)	**0.013**
**Preoperative CEA level (ng/mL) ^b^**	14 (1–1359; 47.5)	12 (2–1312; 60)	0.464
**Extrahepatic disease ^a^**	27 (17.9%)	3 (20.0%)	1.000
**Clinical risk score 3–5 ^a^**	92 (60.9%)	10 (66.7%)	0.875
**Major liver resection ^a^**	**58 (38.4%)**	**15 (100%)**	**<0.001**
Atypical resection	29 (19.2%)	0 (0.0%)	/
Segmentectomy/segmentectomy & atypical resection	3 (2.0%)/14 (9.3%)	0 (0.0%)	/
Bisegmentectomy/bisegmentectomy & atypical resection	10 (6.6%)/37 (24.5%)	0 (0.0%)	/
Right/extended right hepatectomy	28 (18.5%)/6 (4.0%)	13 (86.7%)/2 (13.3%)	/
Left/extended left hepatectomy	6 (4.0%)/3 (2.0%)	0 (0.0%)	/
Trisegmentectomy	6 (4.0%)	0 (0.0%)	**/**
Trisegmentectomy & atypical resection	5 (3.3%)	0 (0.0%)	/
Central resection	4 (2.6%)	0 (0.0%)	/
**R0 resection ^a^**	118 (78.1%)	10 (66.7%)	0.492
**CD ≥ 3a ^a^**	31 (20.5%)	6 (40.0%)	0.161
**90-day mortality ^a^**	7 (4.6%)	2 (13.3%)	0.189
**ISGLS haemorrhage grade C ^a^**	2 (1.3%)	0 (0.0%)	1.000
**ISGLS bile leakage grade C ^a^**	5 (3.3%)	1 (6.7%)	1.000
**ISGLS liver failure – any grade ^a^**	40 (26.5%)	12 (80.0%)	**<0.001**
Grade A ^a^	16 (10.6%)	5 (33.3%)	**0.034**
Grade B ^a^	19 (12.6%)	6 (40.0%)	**0.014**
Grade C ^a^	5 (3.3%)	1 (6.7%)	1.000
**Hospital stay (days) ^b^**	10 (5–63; 7)	14 (8–158; 11)	**0.028**

a Categorical variable reported as n (%), chi-square test;

b continuous variable, non-normal distribution, reported as median (minimum-maximum, interquartile range), Mann-Whitney test;

c categorical variable with more than two groups, reported as n (%), Fisher-Freeman-Halton test;

ASA = American Society of Anaesthesiologists; CEA = carcinoembryonic antigen; CD = Clavien-Dindo classification; ISGLS = International Study Group of Liver Surgery; OSH = one-stage hepatectomy; TSH = two-stage hepatectomy

### Case-control matching

To reduce the bias and equilibrate the number of group members, case-control matching was conducted. Patients from the OSH group (controls) were selected based on the predictors that were statistically significant in bivariate analysis (Table 1): number of liver metastases, neoadjuvant chemotherapy, and extent of liver resection.

Case-control matching returned 14 controls among the OSH group, annotated as case-control matching-OSH. All three variables were statistically significant in the case-control matching model (P < 0.001). The Wilcoxon signed ranks test for the median of differences between before and after matching was insignificant (P = 0.317).

### Analyses after case-control matching

After case-control matching, the TSH and OSH groups were compared (Table 2).

**TABLE 2.** Clinical characteristics of the matched groups

**Clinical characteristics**	**CCM-OSH (n = 14)**	**TSH (n = 15)**	**P value**
**Male sex ^a^**	11(78.6%)	13(86.7%)	1.000
**Age (years) ^b^**	60 (53–78; 13)	64 (45–75; 12)	0.463
**ASA score ≥ 3 ^a^**	2 (14.3%)	2 (13.3%)	1.000
**Primary tumour in right colon ^a^**	2 (14.3%)	1 (6.7%)	1.000
**Primary tumour in left colon ^a^**	10 (71.4%)	6 (40%)	0.125
**Primary tumour in rectum ^a^**	2 (14.3%)	7 (46.7%)	0.063
**> 1 primary tumour ^a^**	0 (0.0%)	1 (6.7%)	1.000
**Primary tumour nodal invasion ^a^**	10 (71.4%)	10 (66.7%)	1.000
**Synchronous liver metastases ^a^**	11(78.6%)	11(73.3%)	1.000
**Number of liver metastases ^b^**	5 (2–12; 6)	5 (2–12; 6)	0.317
**Size of liver metastases (cm) ^b^**	4.6 (1–20; 7)	5 (1.5–11; 5.5)	0.463
**Neoadjuvant chemotherapy ^a^**	13 (92.9%)	13 (86.7%)	1.000
**Preoperative CEA level (ng/mL) ^b^**	9 (1–261; 76)	12 (2–1312; 60)	0.975
**Extrahepatic disease ^a^**	1 (7.1%)	3 (20.0%)	1.000
**Clinical risk score 3–5 ^a^**	13 (92.9%)	10 (66.7%)	0.250
**Major hepatectomy ^a^**	14(100%)	15 (100%)	1.000
Right/extended right hepatectomy	8 (57.1%)/2 (14.3%)	13 (86.7%)/2 (13%)	/
Left hemihepatectomy	1 (7.1%)	0 (0.0%)	/
Trisegmentectomy & atypical resection	3 (21.4%)	0 (0.0%)	/
**R0 resection ^a^**	8 (57.1%)	10 (66.7%)	1.000
**CD ≥ 3a ^a^**	4 (28.6%)	6 (40.0%)	0.688
**90-day mortality ^a^**	1 (7.1%)	2 (13.3%)	1.000
**ISGLS haemorrhage grade C ^a^**	0 (0.0%)	0 (0.0%)	1.000
**ISGLS bile leakage grade C ^a^**	1 (7.1%)	1 (6.7%)	1.000
**ISGLS liver failure – any grade ^a^**	10 (71.4%)	12 (80%)	1.000
Grade A ^a^	4 (28.6%)	5 (33.3%)	1.000
Grade B ^a^	5 (35,7%)	6 (40.0%)	1.000
Grade C ^a^	1 (7.1%)	1 (6.7%)	1.000
**Hospital stay (days) ^b^**	16 (6–63; 13)	14 (8–158; 11)	0.406

a Categorical variable reported as n (%), McNemar test;

b continuous variable, nonnormal distribution, reported as the median (minimum-maximum, interquartile range), Wilcoxon signed ranks test

ASA = American Society of Anaesthesiologists; CCM-OSH = case-control matching one-stage hepatectomy; CEA = carcinoembryonic antigen; CD = Clavien-Dindo classification; ISGLS = International Study Group of Liver Surgery; TSH = two-stage hepatectomy

### Morbidity and mortality

Perioperative morbidity and 90-day mortality rates are provided in Table 1 and Table 2.

**FIGURE 2.**
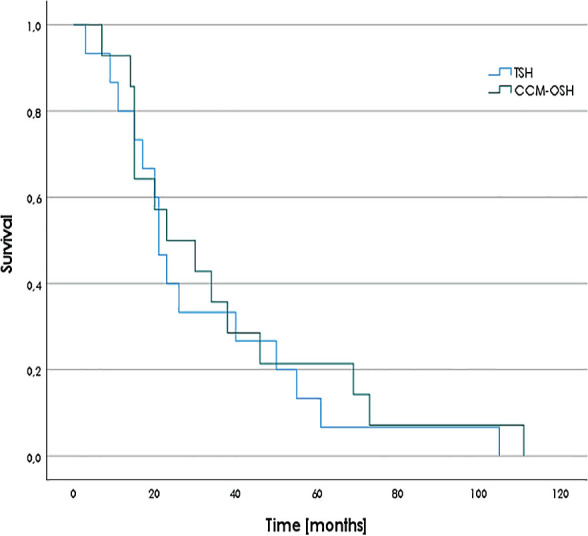
Overall survival after case-control matching (TSH *vs*. case-control matching-OSH groups), P = 0.575. CCM-OSH = case-control matching one-stage hepatectomy; TSH = two-stage hepatectomy

In the OSH group, seven (4.6%) patients died postoperatively. The causes of death were sepsis (n = 1), cardiorespiratory failure (n = 1), multiorgan failure (n = 3), and posthepatectomy liver failure (n = 2). In the TSH group, two (13.3%) patients died postoperatively. One patient suffered from colonic perforation, was reoperated on several times, and died of multiorgan failure. The second patient died of cardiorespiratory failure after acute myocardial infarction. In the case-control matching-OSH group, the patient (7.1%) died of posthepatectomy liver failure.

### Survival analysis

Patients were followed until their death or until 31 December 2022. The median follow-up was 174 (95% CI 113–235) months. A summary of the survival analysis is provided in Table 3 and Figure 2.

**TABLE 3.** Survival analysis

	**Overall**	**OSH (n = 151)**	**TSH (n = 15)**	**P value**
Median OS (months) [95% CI]	35 [30–40]	35 [31–39]	21 [17–25]	0.063
3-year OS	48%	49%	33%	0.107
5-year OS	26%	27%	13%	0.107
RFS (months) [95% CI]	11 [8–14]	11 [9–13]	5 [2–8]	0.138
3-year RFS	14%	15%	13%	0.070
5-year RFS	10%	10%	7%	0.070
**After case-control matching**				
	**Overall**	**CCM-OSH (N=14)**	**TSH (N=15)**	**P value**
Median OS (months) [95% CI]	23 [19–27]	23 [5–41]	21 [17.0–25.0]	0.575
3-year OS	34%	36%	33%	0.743
5-year OS	17%	21%	13%	0.743
RFS (months) [95% CI]	7 [4–10]	8 [1–15]	5 [2–8]	0.888
3-year RFS	14%	14%	13%	0.498
5-year RFS	3%	0%	7%	0.498

CI = confidence interval; CCM = case-control matching; OS = overall survival; OSH = one-stage hepatectomy; RFS = recurrence-free survival; TSH = two-stage hepatectomy

The RFS, median OS, and 3- and 5-year survival rates were 5 months, 21 months, 33% and 13% in the TSH group; 11 months, 35 months, 49% and 27% in the OSH group; and 8 months, 23 months, 36% and 21% in the case-control matching-OSH group, respectively.

## Discussion

The main finding of our research is that the TSH group had a similar survival to that of the case-control matching-OSH group (median OS: 21 *vs*. 23 months), while the major morbidity rate was lower in the case-control matching-OSH group (40% *vs*. 28.6%).

The first TSH was performed on the proposition that a liver resection where some tumour tissue remains in place could be justified if it could be removed by second liver resection.^4^ The time during surgeries was intended for liver hypertrophy, which was enhanced by portal vein embolization or portal vein ligation.^4,17^

Our first TSH was performed in 2005, and 23 patients with the most difficult patterns of colorectal liver metastases were allocated for this demanding treatment (Figure 1).^18,19,20^ Only 15 patients who finished both stages were eligible for this study. This figure is among the lowest, especially compared to the most recent multicentre studies, but close to those in earlier studies.^2^ Regimbeau *et al*. and Chavez *et al*. published large multicentre studies.^5,22^ Their study periods overlap with previous analyses from included centres, as shown in some reviews (Table 4).^21,23^

**TABLE 4.** Literature review of surgical outcomes and survival after two-stage hepatectomy for colorectal liver metastases

**Authors**	**Year**	**Study period**	**N of patients**	**Major liver resection (%)**	**R0 resection (%)**	**Morbidity (%)**	**Mortality (%)**	**Median follow-up (months)**	**Median RFS (months)**	**3-y RFS (%)**	**5-y RFS (%)**	**Median OS (months)**	**3-y OS (%)**	**5-y OS (%)**
Adam *et al*., France^4^	2000	1992–1999	13	62	NR	45	15	22	NR	31	31	44	35	NR
Tanaka *et al*., Japan^17^	2007	1992–2004	22	67	87	23	0	NR	NR	6	NR	NR	33	NR
Wicherts *et al*., France^24^	2008	1992–2007	41	76	NR	59	7	24	NR	26	13	39	60	42
Narita *et al*., France^25^	2011	1996–2009	61	95	NR	54	0	30	NR	15	8	40	59	32
Turrini *et al*., France^26^	2012	2000–2010	34	91	100	20	6	41	NR	24	14	44	59	35
Omichi *et al*., Japan^27^	2022	2013–2019	32	NR	78	22	0	17	6	NR	NR	41	61	NR
The present study, Slovenia	2023	2000–2020	15	100	67	40	13	174	7	13	7	21	33	13
**Multicentre studies**														
Tsai *et al*., USA and Portugal^28^	2010	1994–2008	35	80	NR	26	5	NR	NR	NR	NR	16	58	NR
Regimbeau *et al.* LiverMetSurvey registry^5^	2017	2000–2014	625	NR	58	25	9	84^a^	41^a^	43	23	40^a^	45	23
Petrowsky *et al*. ALPPS registry^29^	2020	2009–2019	510	100	73	21	5	38	11	19	12	37	52	27
Chavez *et al*. Five centres in the USA^22^	2021	2000–2016	196	76	92	23	5	28	NR	19	18	50	64	44

a = mean

ALPPS = associating liver partition and portal vein ligation for staged hepatectomy; N = number; NR = not reported; OS = overall survival; RFS = recurrence-free survival; USA = United States of America

The diversity of first-step procedures challenges further analysis.^30^ Nevertheless, this research adheres to the criteria by Regimbeau *et al*.^5^ except for one case of completed ALPPS.

The clinical characteristics of patients in the OSH and TSH groups differed only in the median number of colorectal liver metastases (3 *vs*. 5) and the rate of chemotherapy treatment (52.3% *vs*. 86.7%). Both characteristics denote an extensive tumour burden in the TSH group.^27^

The concept of parenchyma-sparing liver surgery for colorectal liver metastases was established approximately 30 years ago.^7^ This explains the large group of one-stage hepatectomies (n = 151), even in the case of bilateral colorectal liver metastases, and the lower rate of major hepatectomies in this group (38.4%) (Tables 1 and 2). In the TSH group, the rate of major hepatectomies was 100%, and the difference from the OSH group was statistically significant. The reported rates of major liver resections are given in Table 4.

There was a significant difference in posthepatectomy liver failure when comparing the TSH (80%) and OSH groups (26.5%) (P < 0.001) (Table 1). However, this difference disappeared after case-control matching because the matched group was selected based on neoadjuvant chemotherapy, the extent of liver resection and the number of liver metastases. In the case-control matching-OSH group, 71% of patients had any grade of posthepatectomy liver failure (Table 2), and one patient died of it. However, the reported rates of posthepatectomy liver failure are from 2.6 to 16%.^5,25,27–28^ This wide range may also be due to several definitions of it.^31^ The most commonly used definitions were the following: peak bilirubin > 7 mg/dL^32^, the “50–50” criteria (50% of normal for the prothrombin index and 50 μmol/L for bilirubin on postoperative day 5)^33^, and the definition by the ISGLS used in this study.^14^

The high rate of major morbidity (40% in the TSH group) reflects the burden of demanding two-stage procedures. On the other hand, 4 (28.6%) patients suffered from major morbidity in the case-control matching-OSH group, and the difference between these two groups was statistically insignificant (P = 0.688). The 90-day mortality in our TSH group (13%) did not exceed the reported 15% (Table 4).

TSH aims to improve the survival of patients by resecting all tumoral tissue and enabling sufficient future liver remnant. Our last TSH was performed in 2016, a year before Torzilli *et al*. published results of enhanced OSH as a safe alternative to TSH for multiple bilateral deep-located colorectal liver metastases.^34^

The prognosis was thought to depend on the size and number of colorectal liver metastases.^2^ Whether the resection of colorectal liver metastases could achieve R0, survival was the same regardless of the number of lesions.^2^ However, Fong *et al*. showed that the prognosis depends on the combination of survival factors, i.e., Clinical Risk Score.^9^

There was no significant difference (P = 0.063) in the median OS between the OSH (35 months) and TSH groups (21 months). The rate of R0 resections and the high Clinical Risk Score did not differ. After case-control matching, the OS in the case-control matching-OSH group was 23 months. However, the median OS in recent reports is longer (37–50 months) (Table 4).

Our study showed an insignificant difference in the 3-year RFS between the TSH group (13%) and the case-control matching-OSH group (14%). The 3-year RFS in our study was similar to that reported by Narita *et al*. and Chavez *et al*., but much shorter than the 43% reported by Regimbeau *et al*. (Table 4).^5,22,25^

Limitations of our study could explain these differences in survival. First, this was a single-institution, retrospective study covering a wide period. We had a small group of patients who underwent TSH. Furthermore, the operative technique and use of portal vein embolization have changed over time; thus, it is difficult to apply this study to other modern scenarios. In addition, the interpretation of data and their comparison to other reports was difficult because the TSH group consisted of various first-stage procedures.

To conclude, parenchyma-sparing surgery is a principle of liver surgery for colorectal liver metastases. TSH used to be a safe and favourable therapeutic choice in a select population of patients because it could prevent posthepatectomy liver failure and enable good oncological results. Now, OSH should be preferred whenever feasible because it has lower morbidity and equivalent oncological outcomes as completed TSH.
